# The Effect of the Antioxidant Activity of Plant Extracts on the Properties of Gold Nanoparticles

**DOI:** 10.3390/nano9121655

**Published:** 2019-11-21

**Authors:** Natalia Yu. Stozhko, Maria A. Bukharinova, Ekaterina I. Khamzina, Aleksey V. Tarasov, Marina B. Vidrevich, Khiena Z. Brainina

**Affiliations:** 1 Department of Physics and Chemistry, Research Center of Sensory Technologies, Ural State University of Economics, 8Marta St. 62, 620144 Yekaterinburg, Russia; m.a.buharinova@usue.ru (M.A.B.); xei260296@mail.ru (E.I.K.); tarasov_a.v@bk.ru (A.V.T.); mbv@usue.ru (M.B.V.); baz@usue.ru (K.Z.B.); 2 Department of Analytical Chemistry, Ural Federal University, Mira St. 19, 620002 Yekaterinburg, Russia

**Keywords:** leaf extracts, gold nanoparticles, green synthesis, phytosynthesis, antioxidant activity

## Abstract

Synthesis of gold nanoparticles (phyto-AuNPs) with the use of leaf extracts (phytosynthesis) is based on the concept of Green Chemistry. The present study is conducted to discuss how antioxidant activity (AOA) of extracts from plant leaves impacts on the kinetics of phytosynthesis, the size of the formed nanoparticles, and the stability of their nanosuspensions. Results show that the formation rate of phyto-AuNPs suspensions accelerate due to the increase in the AOA of the extracts. Accompanying the use of transmission electron microscopy (TEM), UV-Vis-spectrophotometry and dynamic light scattering (DLS), it also has been found that higher AOA of the extracts leads to a decrease in the size of phyto-AuNPs, an increase in the fraction of small (*d* ≤ 5 nm), and a decrease in the fraction of large (*d* ≥ 31–50 nm) phyto-AuNPs, as well as an increase in the zeta potential in absolute value. Phyto-AuNPs suspensions synthesized with the use of extracts are more resistant to destabilizing electrolytes and ultrasound, as compared to suspensions synthesized using sodium citrate. Thus, the AOA of the extract is an important parameter for controlling phytosynthesis and predicting the properties of phyto-AuNPs. The proposed approach can be applied to the targeted selection of plant extract that will be used for synthesizing nanoparticles with desired properties.

## 1. Introduction

The unique properties of nanomaterials open up new perspectives in the fields of electronics, catalysis, energy, materials chemistry, sensors, medicine, biology, and agriculture. The properties of nanomaterials are determined by the size, shape and structure of the particles [1,2,3,4], which depend on the synthesis methods. The latter include laser ablation, aerosol technology, lithography, photographic and chemical recovery, the use of ultrasonic fields and ultraviolet radiation. Despite the merits of these methods, they have certain limitations. Physical methods are quite energy consuming and expensive, and chemical methods involve the use of toxic organic solvents, surfactants, strong reducing agents, and production of hazardous by-products. Regarding this, the development of eco-friendly and effective methodologies for the synthesis of nanomaterials is an important task for science and nanotechnology.

The current topic of the last decade has been green nanotechnology, which is a real alternative to hazardous chemical and physical methods and a promising strategy for producing nanomaterials. Green synthesis (terms “biosynthesis”, “phytosynthesis” are also used) is a kind of nanofactory based on simple, single-stage, clean, non-toxic, cost-effective and environmentally friendly approaches. Additionally, the methodology for the green synthesis of nanomaterials is technologically advanced and feasible on an industrial scale, due to the possibility of using widespread raw materials, for example, waste from timber processing industries.

The green synthesis of gold nanoparticles (AuNPs) is of great interest, since their large-scale application in the biomedical sector, the so-called nanomedicine, is planned. This is due to the fact that AuNPs synthesized by green technologies in the size range from 1 to 100 nanometers exhibit antimicrobial [5,6], antifungal [6,7,8], anticancer [7,9,10,11], anti-inflammatory [10,11,12], antioxidant [5,7,8] and immunomodulatory [13] activity. Biocompatibility and low cytotoxicity of AuNPs [14,15,16,17] have been proven and, as a result, they can be used for therapeutic purposes in the treatment and diagnosis of various diseases [18,19,20], for drug [21,22,23] and gene [23] delivery. Additionally, AuNPs obtained by green technologies are used as catalysts in the decomposition of 4-nitrophenol [24,25], as electrode modifiers in the determination of chloramphenicol in milk, honey and eye drops [26], carbendazim in soil [27], lead ions in paints and river waters [28], ecotoxicant hydrazine [29], uric acid in milk and blood serum [30].

Currently, green methods for AuNPs synthesis are being developed using various bio-objects with high reducing ability: bacteria, viruses, fungi and yeast, plants and algae [31,32,33]. A distinctive feature of the nanoparticle’s synthesis with the use of plants (the so-called phytosynthesis) is a higher rate of nanoparticle formation compared to the synthesis rate with the use of microorganisms [34] and the fact that additional reagents are not required [35,36,37,38,39]. Leaf extracts contain a wide range of biomolecules and metabolites, such as terpenoids [19,40,41], vitamins [19,41], polysaccharides [19,41,42], proteins [19,41,42], amino acids [19,41,42], alkaloids [40,42], (poly) phenolic compounds [40,42], aromatic amines [43], tannins [41], saponins [41], ketones [41], aldehydes [41], flavonoids [19,40,41], organic acids [19,42], enzymes [19,42], which act as reducing agents and stabilizers of nanosuspensions in the process of phytosynthesis. It is assumed that the phenolic compounds of plants [44,45], such flavonoids as quercetin, genistein, kaempferol, and proanthocyanidin in particular [46], are primarily responsible for the synthesis of AuNPs.

Fourier transform infrared spectroscopy showed that hydroxyl (–OH) [19,41] and carbonyl (= CO) [45] groups of compounds present in the plants [24,47,48,49,50,51] play a key role in the formation of metal nanoparticles in the photosynthesis process. According to many researchers, the mechanism of phytosynthesis is quite simple: the precursor (Au (III)) is reduced by plant phenolic compounds to AuNPs as a result of the redox reaction with the formation of a phyto-functional coating (phyto-AuNPs) on the surface of the nanoparticles, which protects the particles from aggregation [52,53,54].

Despite significant successes in the field of phytosynthesis of AuNPs and metal nanoparticles in general, there is a problem of controllability of phytosynthesis and prediction of the AuNPs properties. Considering the literature on the phenolic compound’s key role in the phytosynthesis, which are known to have antioxidant properties, we suggest that antioxidant activity (AOA) is a general parameter characterizing the reducing properties of extracts, the kinetics of phytosynthesis, and the properties of AuNPs. Regarding this, the goal of this study is to establish the effect of the AOA of the extracts as model systems on the kinetics of phytosynthesis and the properties of nanoparticles (shape, size) and the stability of AuNPs nanosuspensions.

## 2. Experimental

Section 2 includes the used chemicals and reagents, apparatus, protocols of preparation and AOA estimation of extracts from strawberry, blackcurrant, and gooseberry dried leaves. Synthesis of AuNPs suspensions and methods of AuNPs research also are given in this section.

### 2.1. Chemicals and Reagents

The following chemically pure reagents were used: K_3_[Fe(CN)_6_)] and K_4_[Fe(CN)_6_]∙3H_2_O (AO Reachim Ltd., Moscow, Russia); KCl (JSC ChemReactivSnab, Ufa, Russia), Na_2_HPO_4_∙12H_2_O (CJSC Vekton, St. Peterburg, Russia); KH_2_PO_4_ (NevaReaktiv Ltd., St. Petersburg, Russia), HAuCl_4_ (RPE Tom’analit Ltd., Tomsk, Russia), HCl (NevaReactiv Ltd., St. Petersburg, Russia), NaOH (JSC ChemReactivSnab, Ufa, Russia). All chemicals were used without further purification. Deionized water with a resistivity of 18 MΩ cm was used as a solvent.

### 2.2. Apparatus

To obtain extracts from the plant leaf, a magnetic stirrer with controlled heating (RCT basic IKA–Werke, Staufen, Germany) and a MIKRO 120 centrifuge (Andreas Hettich GmbH, Tuttlingen, Germany) were used. AOA of the extracts was determined using a multifunctional potentiometric analyzer MPA-1 (IVA Ltd., Yekaterinburg, Russia). Spectrophotometric measurements were performed on an ECO-VIEW UV 1200 spectrophotometer (Shanghai Mapada Instruments Co., Ltd., Shanghai, China). High resolution transmission electron microscopy measurements were performed on a JEM-2100 microscope (JEOL Ltd., Tokyo, Japan). Dynamic light scattering measurements were performed on a BrookHaven ZetaPlus analyzer (Brookhaven Instruments Corp., Holtsville, NY, USA). Ultrasonic treatment of AuNPs suspensions was carried out using an Ultrasonic Processor VCX 750 equipped with a titanium stepped microtip 2 mm (Sonics and Materials Inc., Newtown, CT, USA). Deionized water with a resistivity of 18 MΩ cm was obtained on an Akvalab-UVOI-MF-1812 installation (JSC RPC Mediana-Filter, Moscow, Russia).

### 2.3. Preparation of Leaf Extracts

Preparation of extracts from the gooseberry, blackcurrant, and strawberry leaves was carried out in accordance with the procedure [55]. According to the recommendations of Brainina et al. [55], to obtain aqueous solutions with the highest AOA, extraction was carried out at a temperature of 80 °C for 20 min using dried leaves.

Dry leaves were crushed into a powder in a corundum mortar and sieved through a stainless steel sieve with a mesh size of 0.08 mm. Then, 40.0 mg of freshly prepared powder was mixed with 10.0 mL of deionized water at 80 °C. Extraction was carried out under conditions of a controlled temperature (80 °C) and constant stirring for 20 min, which ensured extracts with maximum AOA [55]. After extraction was completed, the mixture, cooled to room temperature was separated into liquid and solid fractions by centrifugation at 10,000 rpm for 5 min. A freshly isolated supernatant was used, hereinafter referred to as a leaf extract.

### 2.4. Determination of AOA of the Leaf Extracts

Determination of AOA of the leaf extracts was performed using a hybrid potentiometric method (HPM) [56]. The analysis procedure and calculations are described in detail in our previous works [55,57]. A 10 mL glass electrochemical cell, a platinum screen-printed electrode (Iva Ltd., Yekaterinburg, Russia) and an EVL-1M3.1 electrode containing 3.5 M KCl (JSC Gomel Plant of Measuring Devices, Gomel, Belarus) were used. After adding 0.2 mL of the leaf extract to 9.8 mL of a solution containing 10 mM K_3_[Fe(CN)_6_], 0.1 mM K_4_[Fe(CN)_6_], 40.8 mM Na_2_HPO_4_ and 25.9 mM KH_2_PO_4_ (pH 7.0), a shift of the indicator (platinum) electrode potential was recorded. The measured value of AOA is presented in mM-eq, i.e., number of moles-equivalents in given volume.

### 2.5. Synthesis of AuNPs

The synthesis of AuNPs suspensions was based on the method used by Turkevich et al. [58] and Frens et al. [59]. Concerning “citrate” synthesis, sodium citrate [60] was used as a reducing agent: 1.5 mL of a 38.8 mM Na_3_Cit solution was added to 15 mL of a boiling solution of 1 mM HAuCl_4_ with vigorous stirring, the synthesis was carried out to obtain a wine-red color of the solution. Thus obtained “citrate” AuNPs (cit-AuNPs) had a spherical shape [58,59,60,61] and a diameter of about 13 nm [60]. Regarding phytosynthesis of AuNPs (phyto-AuNPs), different (0.25, 0.5, 0.75, 1.0, 2.0, 2.0) aliquots of gooseberry, blackcurrant, and strawberry extracts were added to 5 mL of a boiling solution of 1 mM HAuCl_4_ to obtain gb-AuNPs, bc-AuNPs, and sb- AuNPs, respectively. Occurring in all of the above reactions, the reducing agents were in excess with respect to the precursor. A mixture of HAuCl_4_ and the extract was called a “reaction mixture”, and the synthesized aqueous colloidal solutions of AuNPs were called “AuNPs suspensions”. The resulting suspensions of cit-AuNPs and phyto-AuNPs were cooled to room temperature with stirring. It is known that the temperature increase contributes, not only to an increase of the rate of AuNPs formation but, also, to an increase in the spherical AuNPs fraction proportion [62] and a decrease in their size [62,63]. Regarding this, phytosynthesis was carried out at a temperature of 100 °C.

### 2.6. UV-Vis Spectrophotometric Measurements

Absorption of cit-AuNPs and phyto-AuNPs suspensions was recorded relative to a blank sample (deionized water) in the ultraviolet and/or visible part of the spectrum. All suspensions were diluted 3 times with deionized water. Absorption maximum (*A*_max_) of phyto-AuNPs suspensions at a wavelength (*λ*_max_) in the region of 520–560 nm was used to estimate the phytosynthesis rate and diameter of AuNPs. The diameter of AuNPs was calculated by the formula used by Haiss et al. in [64], as shown in Formula 1:

(1) $$d=e^{B_{1}\frac{A_{\max}}{A_{450}\;}-{\;B}_{2}}$$

where d is the diameter of the AuNPs, nm; $A_{\max}$ is the absorption maximum of phyto-AuNPs suspensions, a.u.; *A*_450_ is the absorption at 450 nm, a.u.; B_1_ and B_2_ are fit parameters (B_1_ = 3.55, B_2_ = 3.11) [64].

### 2.7. High Resolution Transmission Electron Microscopy Measurements

Samples for high resolution transmission electron microscopy (HR-TEM, hereinafter TEM) measurements were prepared by immersing a copper mesh in an undiluted suspension of phyto-AuNPs, which was then placed in a vacuum dryer until the water was completely evaporated. TEM measurements were performed with an accelerating voltage of 200 kV and a resolution along the points and along the lines of 0.23 nm and 0.14 nm, respectively. The shape and diameter of phyto-AuNPs were evaluated from the TEM images. Histograms characterizing the size (diameter) distributions of phyto-AuNPs were constructed in Microsoft Excel 2010. The polydispersity index (*PI*) of phyto-AuNPs was calculated in accordance with ISO 22412: 2017 [65]:

(2) $$PI={\left(\frac{s}{\bar{d}}\right)}^{2}$$

where $\bar{d}$ is the average value of a phyto-AuNPs diameter, nm and *s* is the standard deviation, nm.

### 2.8. Dynamic Light Scattering Measurements

Dynamic light scattering (DLS) measurements were performed using Particle Sizing Software (Brookhaven Instruments Corp., Holtsville, NY, USA) (determination of hydrodynamic diameter, *d*_H_) and Zeta Potential Analyzer Software (Brookhaven Instruments Corp., Holtsville, NY, USA) (determination of zeta potential, *ζ*). The determination of *d*_H_, *ζ*, and the polydispersity index of phyto-AuNPs (*PI*) suspensions was carried out on the basis of autocorrelation functions of the scattered radiation intensity.

### 2.9. Assessment of the Aggregate Stability of AuNPs Suspensions

The aggregate stability of phyto-AuNPs and cit-AuNPs suspensions was studied using NaCl as a destabilizing electrolyte [66,67] and ultrasound [68,69]. An NaCl solution was added to 0.6 mL of phyto-AuNPs or cit-AuNPs suspension to obtain suspensions with a concentration of 0.1, 0.2, 0.35, 0.5, 1% NaCl. Ultrasound treatment of 5 mL of phyto-AuNPs’ and cit-AuNPs’ suspension samples was investigated at a frequency of 20 kHz, a power of 750 W, and an amplitude of 20% for 10 min.

### 2.10. Data Treatment

All measurements, except for TEM, were repeated 3 times. Statistical analysis was performed in Microsoft Excel 2010 with an accepted significance level of *α* = 0.05. The results are presented as *X* ± Δ*X*, where *X* is the average value, Δ*X* is the standard deviation.

## 3. Results and Discussions

### 3.1. Characterization of AOA of the Leaf Extracts and Reaction Mixtures by HPM

**Figure 1:**
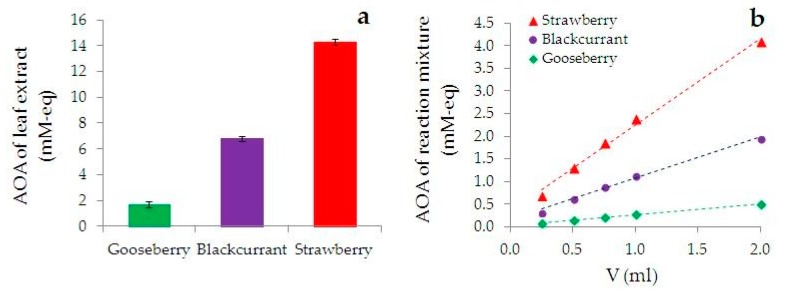
AOA of the leaf extracts (**a**) and the dependence AOA of the reaction mixture (HAuCl_4_ + extract) on an aliquot of the introduced extract (**b**).

Figure 1a shows the results of determining the AOA of the leaf extracts using HPM. The figure shows that the AOA of the extracts increases in the row of gooseberry < blackcurrant < strawberry. The obtained dependences of AOA of the reaction mixtures on the aliquots of gooseberry, blackcurrant, and strawberry extracts introduced into them are shown in Figure 1b. It is seen that an increase in the aliquot of the extract from 0.25 to 2.0 mL leads to an increase in the AOA of the reaction mixture from 0.08 to 0.49 mM-eq in the case of gooseberry extract, from 0.32 to 1.94 mM-eq in the case of blackcurrant extract, and from 0.68 to 4.08 mM-eq in the case of strawberry extract.

### 3.2. Characterization of the Kinetics and Phytosynthesis Completeness Using UV-Vis Spectrophotometry

Kinetics of AuNPs formation during AuCl_4_^–^ precursor reduction by leaf extracts can be considered in the framework of two alternative competing mechanisms, namely, the Finke–Watzky model [70,71] or redox-crystallization model [68,72]. Scientific discussion in favor of any of these kinetics mechanisms is not the subject of this article.

**Figure 2:**
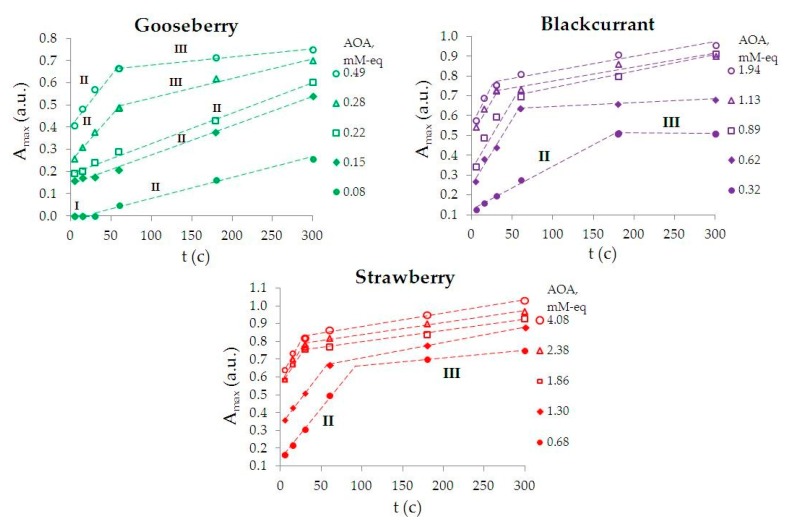
Kinetic curves of the phytosynthesis of phyto-AuNPs suspensions using reaction mixtures containing extracts from gooseberry, blackcurrant and strawberry leaf.

Parallel to Zhong et al. [68] and Radziuk et al. [69], on the kinetic curves (the dependence of absorption maximum of AuNPs suspensions (*A*_max_) on time) obtained in this study (Figure 2), we can distinguish the “induction stage” (I), when AuNPs are not yet formed, the “growth stage” (II), in which there is an active increase in their number, and the “saturation stage” (III), in which the number of AuNPs either does not change at all, or changes slightly, which indicates the completion of the phytosynthesis process.

Seen in Figure 2, the induction stage (I) is observed only on the kinetic curve of the reaction mixtures having AOA = 0.08 mM-eq. When AOA ≥0.15 mM-eq, the induction stage (I) disappears. The saturation stage (III) appears on the kinetic curves at AOA ≥0.28 mM-eq; the earlier, the higher the AOA of the reaction mixture. An increase in AOA of the reaction mixture for each plant leads to an increase in *A*_max_, which corresponds to an increase of the phyto-AuNPs concentration (Bouguer–Lambert–Bera law). The rate of phyto-AuNPs’ suspension formation (*ν*~d*A*_max_/d*t*) at the growth and saturation stages was calculated based on the angular coefficients of the tangents in sections II and III of the kinetic curves in Figure 2, respectively, and are presented in Table 1.

Considering Table 1:

− the rate in section (II) of the kinetic curve is significantly higher than in section (III);
− an increase in AOA of the reaction mixture leads to an increase in the phyto-AuNPs’ suspension formation rate at the growth stage (II), for example, the phyto-AuNPs’ suspension formation rate increases by 4.5 times with an increase in AOA of the reaction mixture of gooseberry from 0.08 to 0.49 mM-eq;
− the higher AOA of the extract from the plant, the greater the rate of the phyto-AuNPs’ suspension formation. Thus, an increase in AOA of the extract in the row of gooseberry (*Ribes uva-crispa*) < blackcurrant (*Ribes nigrum*) < strawberry (*Fragaria vesca*), leads to an increase in the rate of the phyto-AuNPs’ suspension formation. Thus, the rate of formation of AuNPs suspensions in reaction mixtures containing 0.25 mL of gooseberry, currant and strawberry extract is 1.0; 2.2 and 5.7 ms^−1^, respectively.

**Figure 3:**
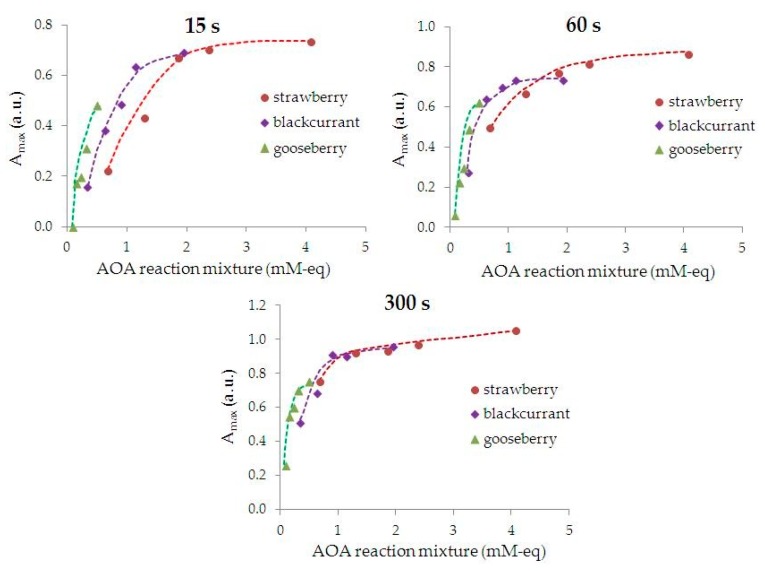
Dependence of phyto-AuNPs suspensions (*A*_max_) absorption maximum on the AOA of the reaction mixture of the leaf extracts from gooseberry, blackcurrant, and strawberry with different durations of phytosynthesis.

Figure 3 shows the dependence of absorption maximum of AuNPs suspensions on AOA of the reaction mixture for different durations of phytosynthesis. Colloidal AuNPs are not formed at AOA ≤ 0.08 mM-eq of reaction mixture and phytosynthesis duration *t* ≤ 15 s. The faster the saturation stage is achieved, the more AOA there is of the reaction mixture.

Based on the analysis of kinetic curves (Figure 2), reaction rates (Table 1), and also the dependence *A*_max_ = ƒ(AOA) (Figure 3), the conclusion can be made that the smaller AOA of the extract, the larger an aliquot of the extract (to create a higher AOA of the reaction mixture) must be used and phytosynthesis should be carried out for a longer time to reach the saturation stage.

To carry out phytosynthesis, the following conditions were set: 5 mL of an aqueous solution of 1 mM HAuCl_4_ + 1 mL of extract with a synthesis time of 300 s. These phytosynthesis conditions for phyto-AuNPs suspensions correspond to saturation stage (III) for all extracts used in this study (Figure 3).

**Figure 4:**
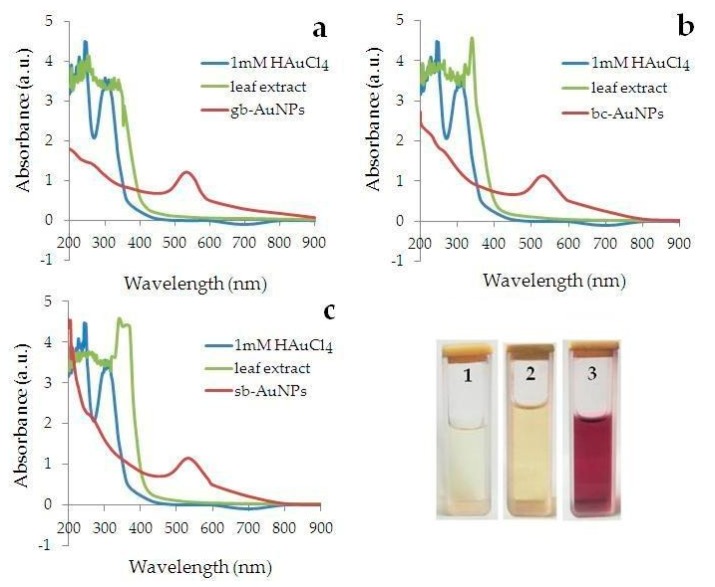
UV-Vis spectra of a 1 mM HAuCl_4_ solution, leaf extracts from gooseberry (**a**), blackcurrant (**b**), strawberry (**c**) and phyto-AuNPs suspensions synthesized for 300 s in a reaction mixture containing 5 mL of 1 mM HAuCl_4_ and 1 mL of leaf extract. Photographs: a solution containing 1 mM HAuCl_4_ (**1**), strawberry extract (**2**) and sb-AuNPs suspension (**3**).

Figure 4 shows the UV-Vis spectra of mixtures containing HAuCl_4_, leaf extracts and phyto-AuNPs suspensions synthesized under the above conditions. Absorption maximum of mixtures are observed at 312 nm, 200–370 nm, and 525–560 nm, respectively. The UV-Vis spectra of phyto-AuNPs suspensions do not contain an absorption maximum characteristic for HAuCl_4_ solution and plant extracts, which indicates the completeness of the phytosynthesis process and the absence of the contribution (errors) of extracts to the absorption maximum of phyto-AuNPs suspensions.

### 3.3. Characteristics of Phyto-AuNPs

**Figure 5:**
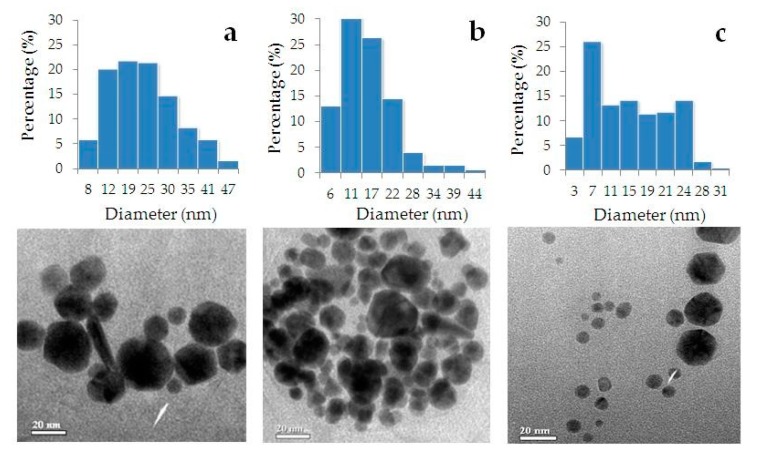
TEM images of phyto-AuNPs synthesized using leaf extracts from gooseberry (**a**), blackcurrant (**b**) and strawberry (**c**), and corresponding histograms of phyto-AuNPs’ size (diameter) distribution. Synthesis conditions: 5 mL of 1 mM HAuCl_4_ + 1 mL of extract, time 300 s.

Figure 5 presents TEM images of phyto-AuNPs synthesized using leaf extracts from gooseberry, blackcurrant, strawberry, and corresponding histograms of the nanoparticle’s size distribution. It is seen that phyto-AuNPs are predominantly spherical. According to Table 2, proportion of spherical particles is approximately the same for gb-AuNPs, bc-AuNPs and sb-AuNPs and is at least 90%.

Table 3 presents the characteristics of phyto-AuNPs obtained by TEM and suspensions of phyto-AuNPs obtained by UV-Vis spectrophotometry and DLS. Table 3 shows that an increase in AOA of the extract in the series of gooseberry, blackcurrant, strawberry leads to a decrease of the average diameter of phyto-AuNPs, (sb-AuNPs < bc-AuNPs < gb-AuNPs). As it will be shown below, TEM data are consistent with data obtained by UV-Vis spectrophotometry and DLS.

The AOA of the extracts’ increase in the row of gooseberry, blackcurrant, and strawberry is accompanied by an increase in the part of small phyto-AuNPs fractions up to 5 nm in diameter and a decrease in the part of large phyto-AuNPs fractions with a diameter of 31–50 nm (Table 4). As expected, all phyto-AuNPs are polydisperse (*PI* > 0.1), which is associated with a sufficient variety of reducing agents present in extracts from plant leaves and their various restorative and stabilizing properties.

**Figure 6:**
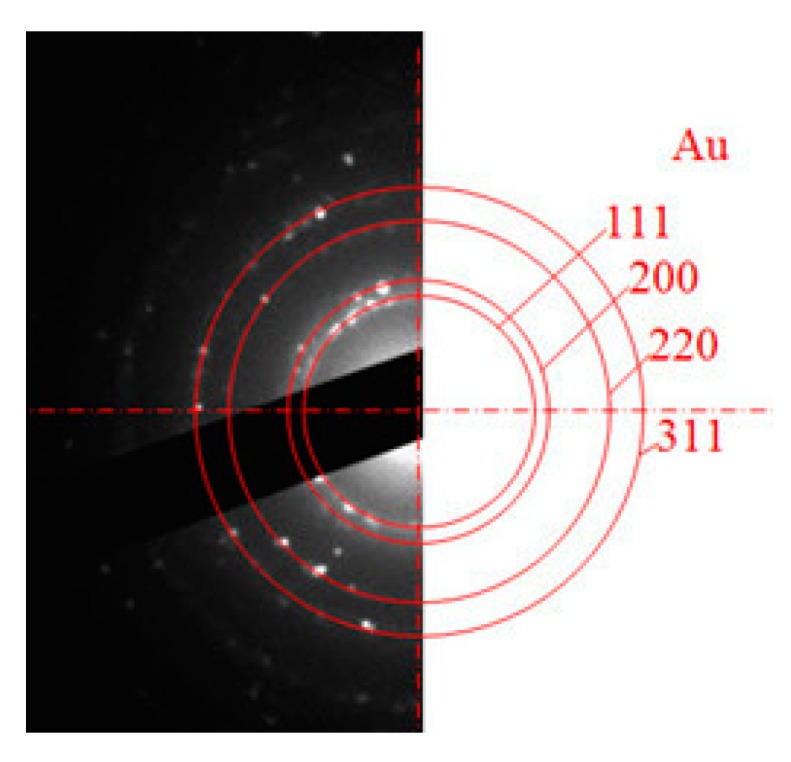
Selected area electron diffraction (SAED) pattern of sb-AuNPs in a 200 kV electron beam.

Figure 6 shows an example of a selected area electron diffraction pattern of sb-AuNPs. An annular diffraction pattern indicates that sb-AuNPs are crystalline. Diffraction rings can be indexed as reflections of the (111), (200), (220) and (311) planes of the face-centered cubic gold lattice.

The AuNPs’ diameter values, calculated by formula 1 using UV-Vis spectrophotometry data and shown in Table 3, demonstrate that, with an increase in AOA of the extracts in the row gooseberry, blackcurrant, and strawberry (Figure 1), the average value of the phyto-AuNPs’ diameter decreases.

**Figure 7:**
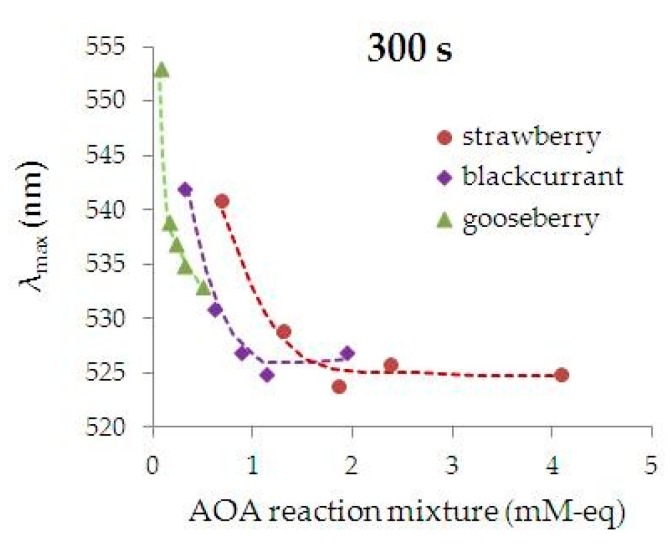
Dependence of the phyto-AuNPs’ suspension absorption maximum wavelength (*λ*_max_) on the AOA of the reaction mixtures containing leaf extracts from gooseberry, blackcurrant, and strawberry. (Synthesis conditions: 5 mL of 1 mM HAuCl_4_ + 1 mL of extract, time 300 s).

Figure 7 shows the dependence of the wavelength of absorption maximum of phyto-AuNPs suspensions (*λ*_max_) on the AOA of the reaction mixture. It follows from Figure 7 that an increase in the AOA of the reaction mixture leads to a shift of *λ*_max_ to a shorter wavelength region of the spectrum, which is a consequence of a decrease in the size of the phyto-AuNPs [73].

**Figure 8:**
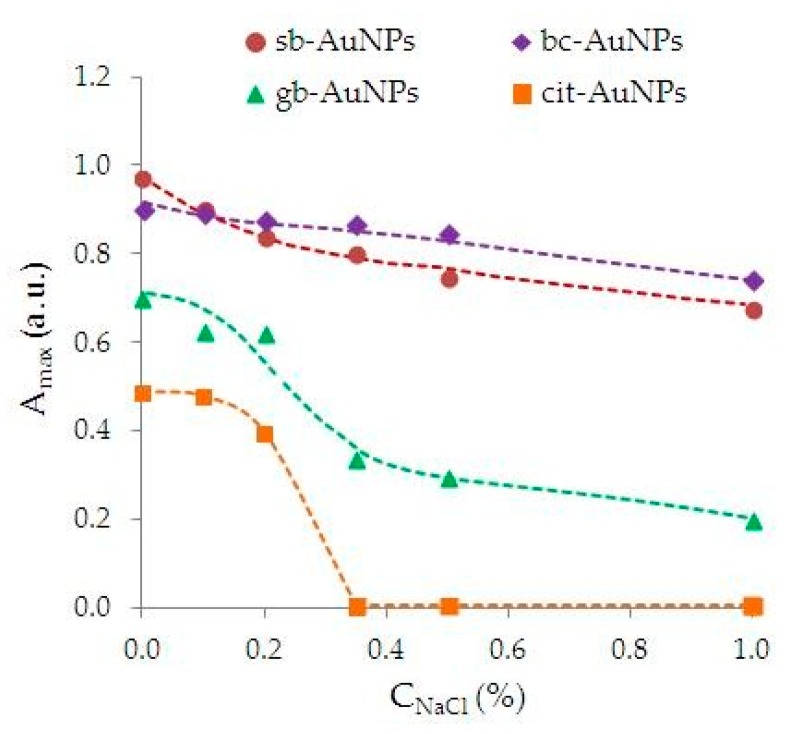
The effect of an NaCl destabilizing electrolyte on the stability of cit-AuNPs and phyto-AuNPs suspensions.

The data demonstrating the aggregate stability of phyto-AuNPs and cit-AuNPs nanosuspensions exposed to NaCl as an electrolyte-destabilizer and ultrasound are presented in Figure 8. Figure 8 shows suspension of cit-AuNPs turned out to be the most unstable to NaCl compared with phyto-AuNPs. A sharp change in absorption maximum of a cit-AuNPs’ suspension begins at a NaCl concentration above 0.2%, and at a concentration of 0.35% NaCl, the absorption maximum is not observed. Suspension of cit-AuNPs becomes almost colorless, and a sediment appears at the bottom of the tube. When NaCl was added to the gb-AuNPs’ suspension synthesized using a leaf extract from gooseberry (with the lowest AOA), a rather sharp decrease in absorption maximum from 0.7 to 0.2 and a color change from burgundy to blue was observed, which indicates particle enlargement. Regarding suspensions bc-AuNPs (leaf extract from blackcurrant) and sb-AuNPs (extract from strawberry), a slight decrease in absorption maximum is observed upon addition of NaCl, their color remains maroon, and the particle size is almost unchanged.

**Figure 9:**
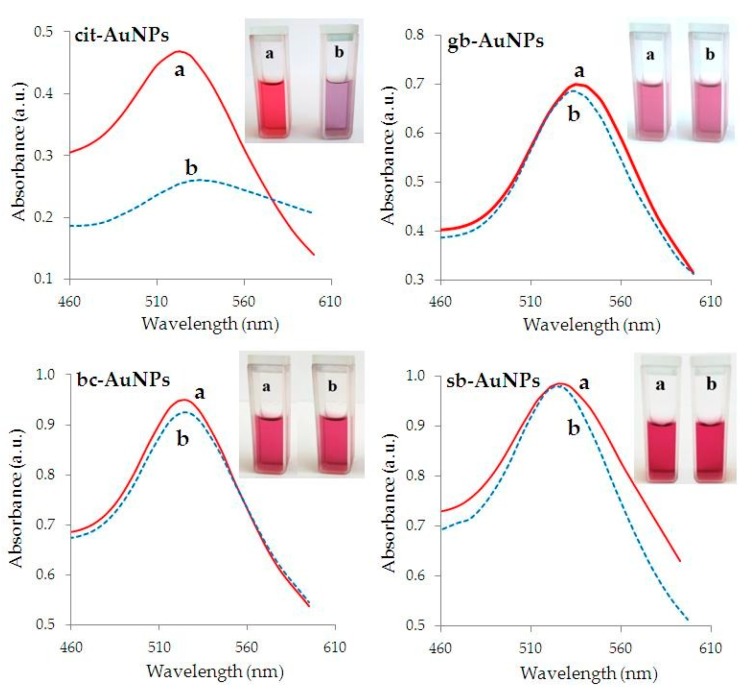
UV-Vis spectra and corresponding photographs of cit-AuNPs, sb-AuNPs, bc-AuNPs and gb-AuNPs suspensions before (**a**) and after (**b**) ultrasound exposure. Parameters of ultrasonic treatment: Ti-microtip, frequency 20 kHz, amplitude 20%, duration 10 min.

Figure 9 presents the optical spectra of cit-AuNPs and phyto-AuNPs suspensions and their photographs before and after a 10-min ultrasonic treatment during which aggregation can occur according to Zhou et al. [72,73]. Figure 9 shows the optical spectra of phyto-AuNPs suspensions practically do not change, in contrast to the cit-AuNPs suspensions; after ultrasonic treatment, it sharply decreases and shifts to the long-wavelength region. The photographs of cit-AuNPs suspensions, before and after ultrasound treatment, demonstrate a clearly visible color change from burgundy to blue/violet, which indicates the aggregation of nanoparticles.

Thus, phyto-AuNPs suspensions are, en masse, more stable than cit-AuNPs suspensions.

Table 3 shows the values of hydrodynamic diameter (*d*_H_), zeta potential (*ζ*) and polydispersity of phyto-AuNPs suspensions (*PI*), measured by the DLS method. It is known that nanosuspensions are stable when the absolute value of *ζ* is ≥25 mV [13,14,74,75]. As can be seen from Table 2, an increase in the AOA of the extract in the row gooseberry, blackcurrant, and strawberry leads to a decrease in *d*_phyto-AuNPs_. Concurrently *ζ* and the stability of phyto-AuNPs suspensions increases. A negative value of *ζ* indicates a negative charge of the potential-determining ions of the double electric layer on phyto-AuNPs. All samples of phyto-AuNPs suspensions are polydisperse (*PI* > 0.1).

An increase in the AOA of the reaction mixture containing strawberry extract in the range of 0.7–4.1 mM-eq leads to a decrease in the hydrodynamic diameter of sb-AuNPs from 55 to 28 nm. Moreover, the stability of sb-AuNPs suspensions does not change much with increasing the AOA of the reaction mixture, and remains high in the considered AOA range, which is obviously caused by a sufficiently high *ζ* (*ζ* = 27–32 mV).

## 4. Conclusions

Green synthesis of nanomaterials is a promising strategy for their production, based on environmentally friendly and cost-effective approaches. Extracts from the dry leaf of plants are good models for studying the features and choosing the conditions of phytosynthesis of nanoparticles. The active component of these reagent models is antioxidant reducing agents that pass into the extract from the plant, the activity of which is determined by the nature of the plant. Metal nanoparticles synthesized using plant extracts have a number of important and valuable properties, such as high anti-cancer, anti-microbial, anti-inflammatory, antioxidant, catalytic activity, etc. and, therefore, are more and more used in various fields of science and practice, and above all in nanomedicine. Despite the growing number of publications on this topic, there are still no common approaches to controlling green synthesis and the properties of phytosynthetic metal nanoparticles. During the present study, using the leaf extracts of gooseberry, blackcurrant and strawberry for the synthesis of gold nanoparticles as an example, we first established the relationship between the kinetics of phytosynthesis and the properties of the synthesized phyto-AuNPs with the AOA of the extracts. It is shown that with an increase in AOA of the leaf extract:

− the rate of phyto-AuNPs formation (phytosynthesis rate) increases;
− the size of phyto-AuNPs decreases;
− the fraction of small phyto-AuNPs (d ≤ 5 nm) increases and the fraction of large phyto-AuNPs (d ≥ 31–50 nm) decreases;
− the stability of phyto-AuNPs suspensions increases.

Thus, the results obtained make it possible to consider the “antioxidant activity” of the plant extract as an important parameter for controlling phytosynthesis and predicting the properties of phyto-AuNPs.

## Figures and Tables

**Table 1:** Phyto-AuNPs’ suspension formation rates (*ν*~d*A*_max_/d*t*) calculated for growth (II) and saturation (III) sections of the kinetic curves in Figure 2.

Extract	Extract Aliquot in the Reaction Mixture, mL	Antioxidant Activity of the Reaction Mixture, mM-eq	*ν*, ms^–1^, (Section II)	*ν*, ms^–1^, (Section III)
Gooseberry (*Ribes uva-crispa*)	0.25	0.08	1.0	–
	0.50	0.15	1.3	–
	0.75	0.22	1.4	–
	1.0	0.28	4.1	0.9
	2.0	0.49	4.5	0.4
Blackcurrant (*Ribes nigrum*)	0.25	0.32	2.2	0
	0.50	0.62	6.3	0.2
	0.75	0.89	6.9	0.8
	1.0	1.13	7.1	0.7
	2.0	1.94	7.9	0.7
Strawberry (*Fragaria vesca*)	0.25	0.68	5.7	0.4
	0.50	1.30	6.0	0.9
	0.75	1.86	7.0	0.6
	1.0	2.38	7.6	0.7
	2.0	4.08	8.2	0.7

**Table 2:** Characteristics of the forms of phyto-AuNPs synthesized using leaf extracts from gooseberry, blackcurrant and strawberry (based on TEM data).

phyto-AuNPs	Percentage (%)			
	Spheres	Triangular Plates	Rhomboid Plates	Rods
gs-AuNPs (*n* = 264)	90.2	6.8	2.6	0.4
bc-AuNPs (*n* = 227)	91.2	5.9	1.9	1.0
sb-AuNPs (*n* = 241)	90.9	5.1	2.3	1.7

*n*—number of particles.

**Table 3:** Characteristics of phyto-AuNPs by TEM and phyto-AuNPs suspensions obtained by UV-Vis spectrophotometry and DLS. (Synthesis conditions: 5 mL of 1 mM HAuCl_4_ + 1 mL of leaf extract, time 300 s). The results are presented as *X* ± Δ*X*, where *X* is the average value and Δ*X* is the standard deviation.

phyto-AuNPs	TEM		UV-Vis-Spectrophotometry	DLS		
	*d*, nm *	*PI*	*d*, nm **	*d*_H_, nm *	*PI*	*ζ*, mV
gb-AuNPs	23 ± 10	0.17	25 ± 3	42 ± 1	0.29	–16 ± 3
bc-AuNPs	15 ± 7	0.21	11 ± 2	38 ± 1	0.33	–17 ± 4
sb-AuNPs	14 ± 7	0.24	10 ± 1	30 ± 1	0.28	–26 ± 1

* Weighted average. ** Number average.

**Table 4:** Characteristics of spherical phyto-AuNPs fractions synthesized using leaf extracts from gooseberry, blackcurrant and strawberry (based on TEM data).

Spherical phyto-AuNPs	Percentage (%)				
	Up to 5 nm	6–10 nm	11–15 nm	16–30 nm	31–50 nm
gs-AuNPs (*n* = 238)	0	13.9	17.6	48.3	20.2
bc-AuNPs (*n* = 201)	1.5	38.8	23.9	33.3	2.5
sb-AuNPs (*n* = 219)	13.7	25.1	20.1	40.6	0.5
